# Application of Carbon-Microsphere-Modified Electrodes for Electrochemistry of Hemoglobin and Electrocatalytic Sensing of Trichloroacetic Acid

**DOI:** 10.3390/s16010006

**Published:** 2015-12-23

**Authors:** Wen-Cheng Wang, Li-Jun Yan, Fan Shi, Xue-Liang Niu, Guo-Lei Huang, Cai-Juan Zheng, Wei Sun

**Affiliations:** Key Laboratory of Tropical Medicinal Plant Chemistry of Ministry of Education, College of Chemistry and Chemical Engineering, Hainan Normal University, Haikou 571158, China; Sdwangwencheng@126.com (W.-C.W.); 18289751071@163.com (L.-J.Y.); shifan0802@126.com (F.S.); xueliangniu@163.com (X.-L.N.); huangguolei1982@163.com (G.-L.H.); sunwei@qust.edu.cn (W.S.)

**Keywords:** hemoglobin, carbon microsphere, direct electrochemistry, trichloroacetic acid

## Abstract

By using the hydrothermal method, carbon microspheres (CMS) were fabricated and used for electrode modification. The characteristics of CMS were investigated using various techniques. The biocompatible sensing platform was built by immobilizing hemoglobin (Hb) on the micrometer-sized CMS-modified electrode with a layer of chitosan membrane. On the cyclic voltammogram, a couple of quasi-reversible cathodic and anodic peaks appeared, showing that direct electrochemistry of Hb with the working electrode was achieved. The catalytic reduction peak currents of the bioelectrode to trichloroacetic acid was established in the linear range of 2.0~70.0 mmol·L^−1^ accompanied by a detection limit of 0.30 mmol·L^−1^ (3σ). The modified electrode displayed favorable sensitivity, good reproducibility and stability, which suggests that CMS is promising for fabricating third-generation bioelectrochemical sensors.

## 1. Introduction

Recently, the electrochemical behavior of proteins has roused great interest, and the results can be applied to the study of electron transfer mechanisms in biosystems and the construction of third-generation electrochemical biosensors or biofuel cells [1,2]. However, the electroactive centers are often buried in the polypeptide chains of redox proteins, which make electron transfer of proteins in a conventional biosensor more difficult [3]. Therefore, various protein-based biosensors have been fabricated for the realization of electrochemical behavior with the usage of multifarious modifiers such as polymers, surfactants and nanosized materials [4,5]. The existence of modifiers can preserve the original structure of redox proteins and their enzymatic activity, which offer a suitable microenvironment for electron transfer between electrode and proteins.

Nanomaterials with multifarious morphologies and unique properties such as excellent biocompatibility and large surface area have been applied to the electrochemistry of protein [6]. Among them, carbon nanomaterials are commonly used due to their excellent electrical conductivity. Various carbon materials have been applied to the investigation of electrochemical behavior of proteins, such as mesoporous carbon [7], carbon nanotubes (CNT) [8], and graphene (GR) [9,10]. Carbon microspheres (CMS) are a kind of carbon material that has been widely investigated. Shin *et al.* proposed a hydrothermal technique for the fabrication of colloidal spheres from aqueous cyclodextrin solution [11]. Sun *et al.* prepared core-shell structures of colloidal carbon spheres with the loading of different nobel-metal nanoparticles [12]. Chen *et al.* synthesized monodispersed carbon spheres from glucose for the supercapacitor [13]. Jin *et al.* proposed a direct hydrocarbon pyrolysis technique for the large-scale synthesis of carbon spheres [14]. The synthesized carbon spheres can be used for the loading of other nanomaterials and applied in different fields such as magnetism, catalysis and biosensor [15,16,17]. However, few reports about the application of CMS in the field of protein electrochemistry have been found.

**Scheme 1:**
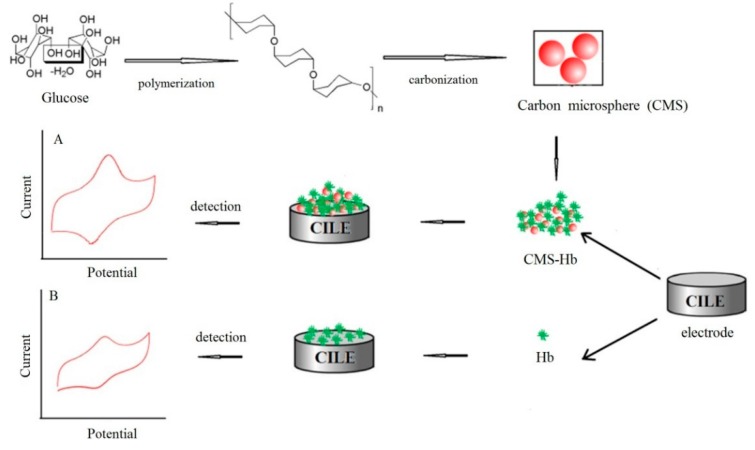
The fabrication process of this Hb modified electrode.

In the present study, CMS was synthesized from glucose using a hydrothermal method and was further applied to the protein electrochemistry. CMS-hemoglobin (Hb) composite was prepared and cast on the ionic liquid modified carbon paste electrode (CILE) surface. Then, CTS were cast for the immobilization of the composite on the electrode surface. The fabrication procedure of this Hb modified electrode is shown in Scheme 1. CILE has exhibited many advantages, such as wide electrochemical windows, high conductivity, good antifouling capability and inherent electrocatalytic ability, which is reported in electroanalysis and electrochemical sensors [18,19]. CTS is an abundant natural cationic biopolymer that is composed of structural repeating units of N-acetyl-glucosamine and glucosamine, which offers a biocompatible microenvironment for the immobilized redox proteins. Therefore, CTS-modified electrodes have been commonly used in electrochemical biosensors [20]. The synthesized CMS were checked using different techniques and exhibited large surface area with porous structure. On CTS/CMS-Hb/CILE the direct electrochemistry of Hb was carried out and the electrocatalysis of trichloroacetic acid (TCA) was achieved, demonstrating the potential applications of this electrochemical sensor.

## 2. Experimental

### 2.1. Reagents

Glucose (Beijing Chem. Reagent Factory, Beijing, China), Hb (Sinopharm. Chem. Reagent, Shanghai, China), 1-butylpyridinium hexafluorophosphate (BPPF_6_ > 99%, Lanzhou Greenchem. CAS., Lanzhou, China), chitosan (CTS, Dalian Xindie Ltd., Dalian, China) graphite powder (30 μm, Shanghai Colloid Chem., Shanghai, China) and TCA (Tianjin Kemiou Chem. Ltd., Tianjin, China).The experiments were conducted in 0.1 mol·L^−1^ phosphate buffer saline (PBS) at room temperature (25 ± 1 °C), which was bubbled with pure N_2_ for half hour before experiments to deoxygenate and keep under N_2_ atmosphere during the electrochemical measurements. Ultrapure water and other chemicals (analytical reagent grade) were used in the experiments.

### 2.2. Apparatus

CHI 660D workstation (Shanghai CH Instrument, Shanghai, China); Nicolet 6700 FT-IR spectrophotometer (Thermo Fisher Scientific Inc., Waltham, Massachusetts, USA); TU-1901 double beam UV-Visible spectrophotometer (Beijing General Instrument Ltd. Co., Beijing, China); Renishaw InVia Raman microspectrometer using 532 nm lasers (Renishaw Plc., London, UK); JSM-7100F scanning electron microscope (Japan Electron Co., Tokyo, Japan); FEI Tecnai G2 F20 microscope (FEI, Hillsborough, Oregon, USA) with a field-emission gun operating at 200 kV; D8 advance X-ray diffractometer (Germany Bruker Co., Karlsruhe, Germany). A three-electrode system was used with a modified electrode (the working electrode), platinum wire (the counter electrode) and saturated calomel electrode (SCE, the reference electrode).

### 2.3. Synthesis of CMS

CMS was fabricated on the basis of the previous report [12]. In general, 8.0 g of glucose was added to 40 mL water with ultrasonic agitation for 2 min to get a colorless solution, which was positioned in teflon-sealed autoclave and kept for 4 h in 180 °C. The brown or black product was segregated by centrifugal separation, cleaned by 3 cycles of centrifugal separation/washing/re-dispersion in water and alcohol, and dehydrated at 80 °C for over 4 h to get black powder. A 1.0 mg·mL^−1^ CMS liquor was produced by redispersing CMS into the water with ultrasonic agitation for 3 h to get a homogeneous suspension solution.

### 2.4. Electrode Fabrication

CILE was manufactured as described previously [21]. In brief, graphite powder were mixed thoroughly with ionic liquid BPPF_6_ at 3/1 (w/w) in a mortar, then the paste was inserted into a glass tube (Ф = 4.0 × 10^−3^ m). Prior to use, CILE was smoothed to get a mirror-like surface.

The step of fabricating the modified electrode was as follows. A 6.0 μL of 0.5 mg·mL^−1^ CMS and 15.0 mg·mL^−1^ Hb mixture solution was directly cast on the CILE surface with a 10.0 μL microsyringe. Then, the working electrode (CTS/CMS-Hb/CILE) was fabricated by spreading 5.0 μL of 1.0 mg·mL^−1^ chitosan (in 1.0% HOAC) solution evenly onto the CMS-Hb/CILE surface. A uniform film on the modified electrode was formed by covering a beaker to alleviate the evaporated solution. The preparation processes of CTS/Hb/CILE, CTS/CMS/CILE, CTS/CILE *etc*., were parallel to that of CTS/CMS-Hb/CILE.

## 3. Results and Discussion

### 3.1. Morphological and Structural Characterization

**Figure 1:**
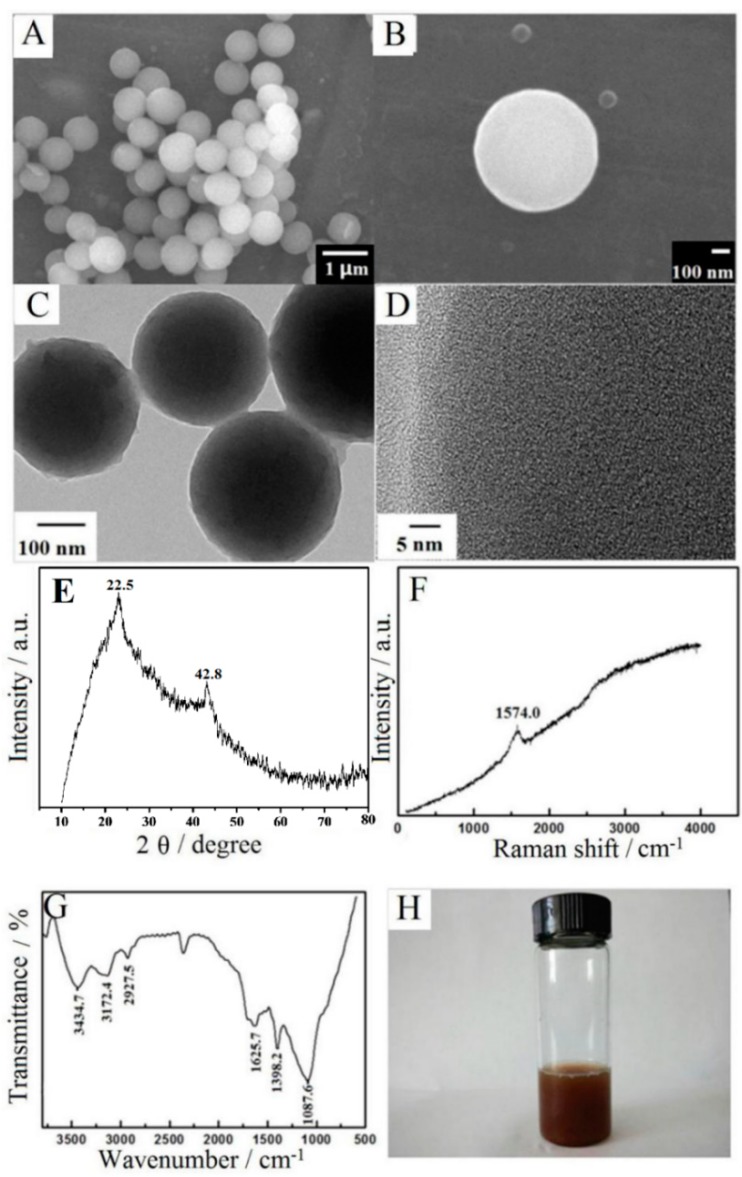
(**A**,**B**) SEM; (**C**,**D**) HRTEM; (**E**) XRD; (**F**) Raman spectrum; (**G**) FT-IR spectrum of CMS; (**H**) Photo of 1.0 mg·mL^−1^ CMS solution that kept for 2 days.

SEM images of CMS with different magnification are shown in Figure 1A,B. It can be seen that the synthesized CMS had an average diameter of 600 nm, which was in good agreement with the reference [12]. TEM images of CMS at different magnitude were shown as Figure 1C,D, which exhibited that the solid structure of the surface had many nanosized pores. These pores on the surface of CMS could be attributed to the release of unreacted organic compounds such as oligosaccharides that been washed by water and alcohol. The existence of pores could offer large surface area and more reactive species for aiding in penetration and adsorption. Based on the reference [12], this synthesis process of CMS is a completely environmental-friendly procedure without the employment of any poisonous reagents, surfactants or organic reagent. Therefore the as-prepared CMS are nontoxic, with potential applications in biosensors or bioelectrochemistry.

The graphitization degrees of CMS were further checked by XRD and Raman spectroscopy. As shown in Figure 1E, two peaks at 22.5° and 42.8° appeared on XRD, which could be specified as the typical graphitic (002) and (100) planes. The broadening of these two peaks suggests that the degree of graphitization was low and the amorphous carbon was possibly subsistent [14]. Figure 1F shows the Raman spectrum of CMS, which had one significant peak at 1574.0 cm^−1^. This peak corresponds to the G-band that is attributed to the ordered graphite structure [22].

The functional groups on CMS were further characterized by an FT-IR spectrum with the result shown in Figure 1G. The O–H bond stretching vibration led to the strong characteristic peak at 3434.7 cm^–1^. The C–H bond stretching vibration led to the weak peaks at 3172.4 cm^–1^ and 2927.5 cm^–1^. The absorption bands at 1398.2 cm^–1^ corresponded to C-C stretching vibration and that of 1625.7 cm^–1^ was the carboxyl groups (C=O) stretching vibration [13]. Therefore, on the surface of CMS, many functional groups were present, which resulted in the hydrophilicity and stability of CMS in aqueous solution. As shown in Figure 1H, the CMS solution could remain stable for 2 days without congregation, indicating that CMS was stable in water and could be used for bioapplications.

### 3.2. Spectroscopic Results

**Figure 2:**
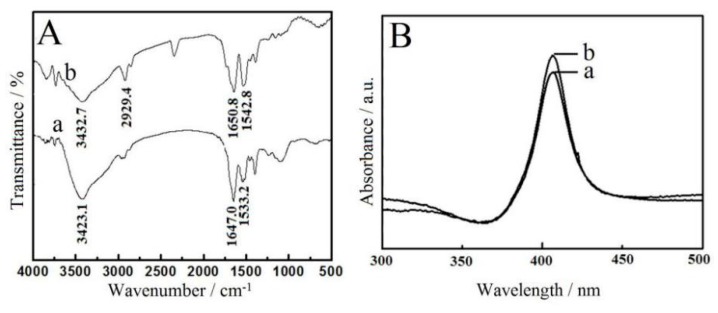
(**A**) FT-IR spectra of Hb (**a**) and CMS-Hb (**b**); (**B**) UV-Vis spectra of Hb (**a**) and CMS-Hb (**b**).

FT-IR spectroscopy was employed to examine the integrity of the structure and structural changes of the proteins [23]. Detailed data of the polypeptide chain could be determined from the shapes of amide I and II [24]. The amide I (1650.8 cm^−1^) and II band (1542.8 cm^−1^) of Hb after being mixed with CMS is shown in Figure 2Ab, which had less difference compared with amide I (1647.0 cm^−1^) and II (1533.2 cm^−1^) bands of the native Hb (Figure 2Aa). The results indicated that the original structure of Hb after being mixed with CMS was unchanged. UV-Vis adsorption spectroscopy is another useful tool to monitor the conformation change of heme proteins [25]. The Hb molecules had a characteristic band (406.0 nm) in pH 3.0 PBS (Figure 2Ba), which was the same as that of CMS-Hb (Figure 2Bb), meaning Hb molecules retained their original conformation in the CMS-Hb solution. All the spectroscopic research indicated that the biocompatibility of CMS and Hb kept the fundamental active conformation of the original structure after being mixed with CMS.

### 3.3. Electrochemical Characterization

**Figure 3:**
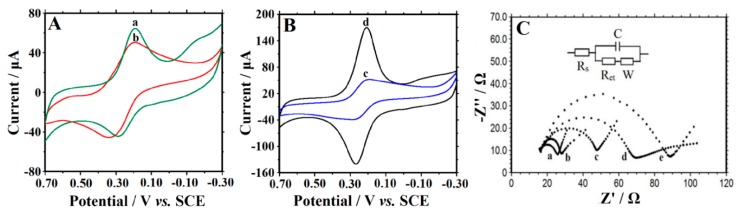
CV of (**A**) CILE (a), CTS/CILE (b); (**B**) CTS/Hb/CILE (c), CTS/CMS-Hb/CILE (d) in a 10.0 mmol·L^−1^ [Fe(CN)_6_]^3−/4−^ and 0.1 mol·L^−1^ KCl solution, scan rate:0.1 V·s^−1^; (**C**) EIS of (**a**) CTS/CMS/CILE; (**b**) CTS/CMS-Hb/CILE; (**c**) CILE; (**d**) CTS/CILE and (**e**) CTS/Hb/CILE with the frequencies ranging from 10^5^ to 10^−1^ Hz. (Inset is the Randles circuit model in the cell).

Electrochemical behaviors were verified by cyclic voltammetry in the 0.1 mol·L^−1^ KCl and 10.0 mmol·L^−1^ [Fe(CN)_6_]^3−/4−^ mixture solution and the results are shown in Figure 3A,B. On CILE (Figure 3Aa) a couple of symmetric redox peaks was shown and it was the representative response of CILE. The electrochemical response of CTS/CILE (Figure 3Ab) was weaker than that of CILE, which was attributed to the existence of unconductive CTS on the surface of electrode impeding the electron transfer. The smallest redox peak currents appeared on CTS/Hb/CILE (Figure 3Bc), proving that the existence of Hb molecules on the electrode surface further impeded the electron transfer. However, on CTS/CMS-Hb/CILE (Figure 3Bd) the redox peak currents increased with the highest value, which was ascribed to the existence of CMS that promoted the electron transfer rate of [Fe(CN)_6_]^3−/4−^ with increased responses.

Figure 3C shows the electrochemical impedance spectroscopy (EIS) of different electrodes, which was employed to investigate the interfacial information [26]. The electron transfer resistance (*R_et_*) value of CILE was found to be 37.97 Ω (curve c) and that of CTS/CILE increased to 54.75 Ω (curve d), proving that the interfacial resistance was increased with the existence of unconductive CTS film. On CTS/Hb/CILE (curve e) the *R_et_* was 82.83 Ω, which could be ascribed to the existence of Hb further increasing the interfacial resistance. While on CTS/CMS/CILE (curve a) and CTS/CMS-Hb/CILE (curve b) the *R_et_* decreased to 10.76 Ω and 15.25 Ω, respectively, showing that the existence of CMS decreased the resistance. CMS is a carbon material with good conductivity that can quicken the electron transfer.

### 3.4. Direct Electrochemistry of Hb

**Figure 4:**
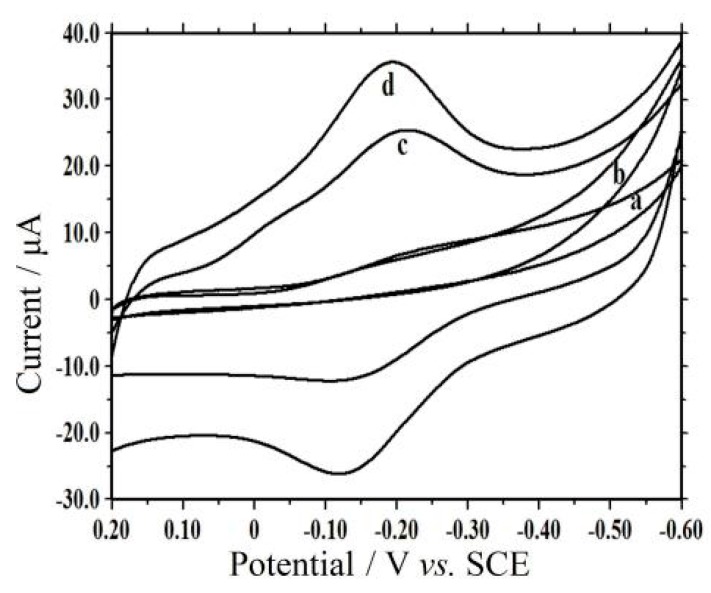
CV of (**a**) CILE; (**b**) CTS/CMS/CILE; (**c**) CTS/Hb/CILE and (**d**) CTS/CMS-Hb/CILE in pH 3.0 PBS, scan rate: 0.1 V·s^−1^.

Figure 4 showed the electrochemical behaviors of different modified electrodes in PBS (pH 3.0). No redox peaks were found on CTS/CILE (curve a) and CTS/CMS/CILE (curve b). With a layer of IL present on the surface, CILE exhibits good conductivity and a biocompatible surface [9], and a couple of asymmetric redox peaks were found on CTS/Hb/CILE (curve c), showing that direct electron transfer between Hb and CILE was realized. On CTS/CMS-Hb/CILE the redox peaks increased greatly (curve d), which remained nearly unchanged at multi-scan cyclic voltammogram. The mixture of CMS with Hb on the electrode can form a biocomposite with good stability, which is suitable for accelerating electron transfer from the electroactive center of Hb to the electrode. The cathodic (Epc) and the anodic (Epa) peak potential were found to be −0.140 V and −0.227 V with the ΔE_p_ as 87 mV. The formal peak potential (E^0′^) was calculated as −0.184 V (*vs*. SCE). The ratio of the cathodic (Ipc) and the anodic (Ipa) peak current was found to be 1.06. To explore the best CV responses of Hb on CTS/CMS-Hb/CILE, the amount of CMS cast on the electrode was optimized in the control experiments with a concentration range of 0.05 to 5.0 mg·mL^−1^. The highest redox currents appeared at 0.5 mg·mL^−1^ CMS, which was used for electrode modification.

### 3.5. Electrochemical Investigation

**Figure 5:**
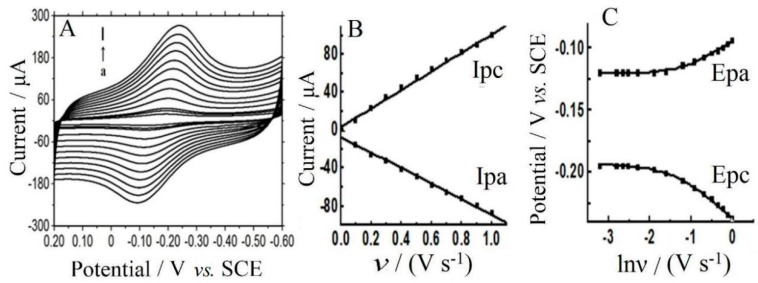
(**A**) Effect of scan rates (*υ*) (from a→l as 50, 80, 100, 200, 300, 400, 500, 600, 700, 800, 900, 1000 mV·s^−1^) on electrochemical responses of CTS/CMS-Hb/CILE in pH 3.0 PBS; (**B**) Plot of the redox peak currents against *υ*; (**C**) plot of the redox peak potentials against ln*υ*.

As shown in Figure 5A, the influence of scan rate (*υ*) on voltammetric responses of CTS/CMS-Hb/CILE was checked. A couple of redox peak appeared at different scan rates with the regression equations of peak currents and *υ* as Ipc(μA) = 92.5·υ (V·s^−1^) + 6.9 (n = 10, γ = 0.999) and Ipa(μA) = −78.0 *υ*·(V·s^−1^) − 10.0 (n = 10, *γ* = 0.999) (Figure 5B), indicating a surface-controlled thin-layer electrode behavior. The integration of the redox peak in cyclic voltammograms can count the surface coverage (Γ*) of electroactive Hb by the formula (Γ* = Q/nAF). By subtracting the background current, the peak currents of Hb were integrated with the Γ* value found to be 9.06 × 10^−10^ mol·cm^−2^, which was larger than the theoretical monolayer coverage (1.89 × 10^−11^ mol·cm^−2^) [27]. The fraction of electroactive Hb among the total Hb (1.20 × 10^−8^ mol·cm^−2^) was calculated as 7.6%. As shown in Figure 5C, the increase in *v* resulted in the change of the redox peak potentials and the relationships of Ep with ln*υ* at high scan rate range were built. Two regression equations were got as Epc(V) = −0.03 ln*υ* − 0.23 (n = 7, *γ* = 0.997) and Epa(V) = 0.02 ln*υ* − 0.10 (n = 7, *γ* = 0.993). Based on the Laviron’s method [28], the kinetic of the redox reaction could be calculated with the electron transfer coefficient (*α*) and electron transfer rate constant (*k_s_*) as 0.444 and 0.946 s^−1^. The *k_s_* value was larger than those of Nafion/Hb-graphene oxide-IL/CILE (0.92 s^−1^) [29], GR/Fe_3_O_4_/Hb/GCE (0.30 s^−1^) [30], Nafion/GR-TiO_2_-Hb/CILE (0.65 s^−1^) [31] and Hb-IL-MWCNT-CPE (0.84 s^−1^) [32], indicating a relatively high rate of electron transfer. Also the *ks* value of CTS/Hb/CILE was calculated as 0.389 s^−1^ with the same method, which was less than that of CTS/CMS-Hb/CILE (0.946 s^−1^). Therefore the presence of CMS with high conductivity provides an enhanced electron transfer reaction for Hb.

**Figure 6:**
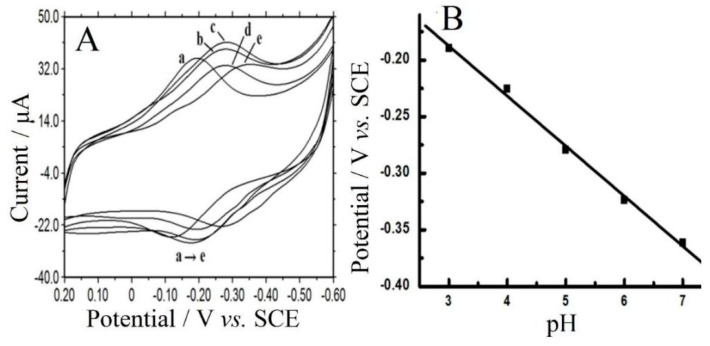
(**A**) CV of CTS/CMS-Hb/CILE in 0.1 mol·L^−1^ different pH PBS (from a→e: 3.0, 4.0, 5.0, 6.0, 7.0), scan rate: 0.1 V·s^−1^; (**B**) the relationship of E^0^′ with pH.

The influence of buffer pH on electrochemical behaviors of CTS/CMS-Hb/CILE was studied in different PBS and the results are displayed in Figure 6A. The linear equation between *E^0′^* and pH was found to be E^0′^(mV) = −44.25·pH − 54.73 (γ = 0.998). The slope value (−44.25 mV·pH^−1^) was smaller than the theoretical value (−59.0 mV·pH^−1^) for a one-proton and one-electron transfer process [33,34]. At pH 3.0 buffer solution, the largest redox peak currents appeared, and it was selected for the electrochemical experiments.

### 3.6. Electrocatalysis

**Figure 7:**
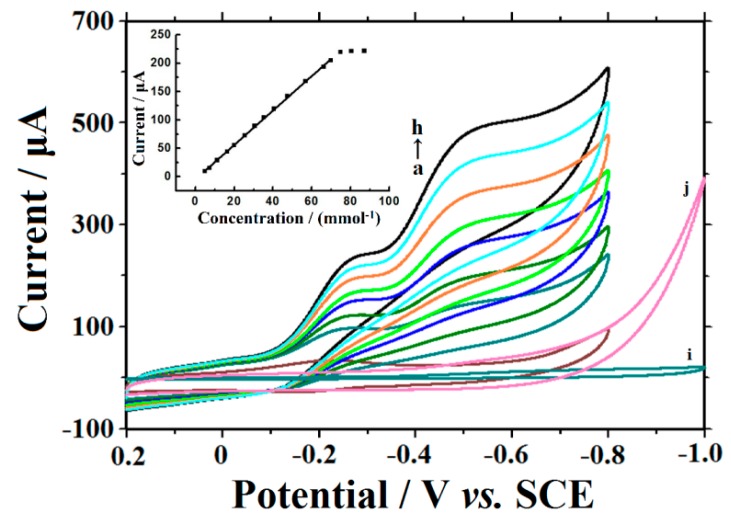
CV of CTS/CMS-Hb/CILE with 0, 15.0, 25.0, 35.0, 45.0, 55.0, 65.0, 70.0 mmol·L^−1^ TCA (curves a→h) and of CTS/CMS/CILE with of 0, 80.0 mmol·L^−1^ TCA (curves i and j), scan rate: 0.1 V·s^−1^ (Inset was the linearity of catalytic reduction currents and TCA concentration).

The redox-protein-based biosensor exhibits excellent electrocatalytic ability for TCA, which is an important analytical target in biochemistry and environmental chemistry. As shown in Figure 7, when various concentrations of TCA were analyzed by CTS/CMS-Hb/CILE, the reduction peak current increased at −0.243 V without oxidation peak (curves a-h). With the further increase of the TCA concentration another reduction peak appeared at −0.515 V, indicated that di- and mono-chloroacetic acid might be dechlorinated by the formation of Hb [Hb Fe(I)] after TCA dechlorination with Hb Fe(II) [35]. On CTS/CMS/CILE direct electroreduction of TCA was studied with the potential negatively than −0.8 V (curves i, j). Therefore the reduction potential of TCA was reduced due to the existence of Hb. The reaction of electrocatalysis could be inferred from the following equations [35]:

$$\begin{array}{l} \text{HbFe(III)}\;+\;\text{e}\;\to \;\text{HbFe(II)}\\ 2\text{HbFe(II)}\;+\;{\text{H}}^{+}\;+\;{\text{Cl}}_{3}\text{CCOOH}\;\to \;2\text{HbFe(III)}\;+\;{\text{Cl}}^{-}\;+\;{\text{Cl}}_{2}\text{CHCOOH}\\ \text{HbFe(II)}\;+\;\text{e}\;\to \;\text{HbFe(I)}\\ 2\text{HbFe(I)}\;+\;{\text{H}}^{+}\;+\;{\text{Cl}}_{2}\text{CHCOOH}\;\to \;2\text{HbFe(II)}\;+\;{\text{Cl}}^{-}\;+\;\text{Cl}{\text{CH}}_{2}\text{COOH}\\ 2\text{HbFe(I)}\;+\;{\text{H}}^{+}\;+\;\text{Cl}{\text{CH}}_{2}\text{COOH}\;\to \;2\text{Hb}\;\text{Fe(II)}\;+\;{\text{Cl}}^{-}\;+\;{\text{CH}}_{3}\text{COOH} \end{array}$$

The catalytic cathodic peak current at −0.243 V depended linearly on the TCA concentration with the equation of Iss(μA) = 3.04 C (mmol·L^−1^) − 4.89 (n = 13, *γ* = 0.999). The linear range and the detection limit were obtained as 2.0 ~ 70.0 mmol·L^−1^ and 0.30 mmol·L^−1^ (3σ), respectively. The cathodic peak current reached a stable value when the TCA concentration exceeded 70.0 mmol·L^−1^, indicating a typical Michaelis-Menten kinetic process. The Lineweaver-Burk equation of 1/I_ss_ = (1/I_max_) (*1 + K_M_^app^/C*) [36] was used to calculate the apparent Michaelis-Menten constant (*K_M_^app^*) at a value of 1.60 mmol·L^−1^, which was smaller than those of published values [27,37,38,39]. The low KMapp value indicated that Hb entrapped on the electrode retained its bioactivity and had a much higher biological affinity to TCA.

### 3.7. Analytical Application

To verify its application, CTS/CMS-Hb/CILE was used for determining of TCA in lab water with the standard addition method. As shown in Table 1, no TCA were found in the water specimens and the recovery was in the range of 97.00%–104.00%. Therefore this Hb-modified electrode could be used for water specimen detection.

**Table 1:** Detection results of TCA in the lab water specimen (n = 3).

Specimen	Found (mmol·L^−1^)	Added (mmol·L^−1^)	Total (mmol·L^−1^)	Recovery (%)	RSD (%)
water	0	2.00	2.08	104.00	3.06
		4.00	3.88	97.00	3.10
		6.00	6.12	102.00	2.98

### 3.8. Stability and Reproducibility

The reproducibility was studied by applying six Hb-modified electrodes to the determination of 10.0 mmol·L^−1^ TCA independently. An acceptable relative standard deviation (RSD) of 2.15% was determined. CTS/CMS-Hb/CILE was put into a 4 °C refrigerator for a certain period to check the storing stability. Every 5 days, the peak response of CTS/CMS-Hb/CILE to 10.0 mmol·L^−1^ TCA was tested, which decreased by 3.1% after 10 days and 7.5% for 25-days storage, proving the relative good stability of CTS/CMS-Hb/CILE.

## 4. Conclusions

CMS was prepared using a hydrothermal method and direct electrochemistry of Hb was carried out on CMS-modified CILE. A couple of well-defined redox peaks could be seen on CTS/CMS-Hb/CILE, indicating the acceleration of direct electron transfer of Hb due to high conductivity, large surface area and good biocompatibility of CMS. The immobilized Hb molecules kept their original structure and exerted good electrocatalytic ability for the reduction of TCA. As compared with other types of redox-protein-modified electrodes for TCA detection [29,31,37,38,39], this Hb-based electrode exhibited advantages such as favorable sensitivity, linear range, detection limit, stability and reproducibility. Hence, CMS has potential for the construction of third-generation redox-protein-based bioelectrochemical sensors.

## Abbreviations

The abbreviations involved in the paper.

**Sigillum**	**Full Caption**	**Sigillum**	**Full Caption**
BPPF_6_	1-butylpyridinium hexafluorophosphate	HOAC	acetic acid
CILE	carbon ionic liquid electrode	IL	ionic liquid
CPE	carbon paste electrode	Ipa	anodic peak current
CMS	carbon microsphere	Ipc	cathodic peak current
CTS	chitosan	*K_M_^app^*	apparent michaelis-menten constant
CV	cyclic voltammograms	MWCNT	multi-walled carbon nanotube
EIS	electrochemical impedance spectroscopy	PBS	phosphate buffer solutions
E^0′^	formal peak potential	Ret	the electron transfer resistance
Epa	anodic peak potential	RSD	the relative standard deviation
Epc	cathodic peak potential	SEM	scanning electron microscopy
GOD	glucose oxidase	TCA	trichloroacetic acid
GR	graphene	TEM	transmission electron microscopy
GCE	glass carbon electrode	XRD	X-ray diffraction
Hb	hemoglobin	*Γ**	surface coverage
